# Graduate Study in London

**Published:** 1908-02-22

**Authors:** 


### HOSPITAL ADMINISTRATION.

### CONSTRUCTION AND ECONOMICS.

### GRADUATE STUDY IN LONDON.

### II.—THE POSSIBILITIES OF THE POLYCLINIC.

In a previous article (The Hospital, Feb. 15, p. 533) we have endeavoured to outline the scope of the work which is at present being done at the Polyclinic, and to show how widely the general practitioner has availed himself of the advantages offered by that institution. It remains to consider how far these advantages are capable of extension, and in what way, or by what means, the work that is done may be made to appeal even more widely to the profession than it does at present. At a first glance it seems an invidious task to criticise so excellently worked an institution, which has proved its merit by sheer, unostentatious perseverance in the face of great difficulties. For all that no one who has thoroughly considered the subject of post-graduate study, both here where the movement in favour of it is as yet in its infancy, and elsewhere where it has established itself on a firm basis, can fail to detect in the system on which the Polyclinic and the few other institutions that are modelled on analogous lines are run, certain defects and certain deficiencies. To point out these defects and to suggest means whereby they may be overcome and remedied is a task that the critic, if he wishes to be something more than merely destructive or laudatory, cannot honestly shirk.

The great defect of post-graduate study in London as it exists at present is in the fact that there is no effective clinical teaching in its strict sense. The Polyclinic has no beds; it is not a hospital, but a medical school. It is true there are institutions, such as the West London Hospital, the London School of Clinical Medicine, the Prince of Wales's General Hospital at Tottenham, and the Mount Vernon Hospital, which possess their own beds and which cater specially for the graduated student in medicine. It is true also that there exists the excellent London Post-graduate Association, which offers exceptional facilities for clinical study to qualified medical men. Membership of this Association gives the graduate the privilege of walking the wards at practically all our general and special metropolitan hospitals. In the circumstances, therefore, it may be argued that the statement above made is inaccurate and exaggerated. Yet a moment's consideration will bear out its truth. The medical graduate who wishes to avail himself of the advantages offered by a post-graduate course stands on an entirely different footing from the student who has yet to qualify. Unlike the latter, he has learnt—or ought to have learnt—most of what the wards of the general hospital can tell him, just as he has learnt most of what can be taught in general lectures such as those that are given for the students' benefit. He comes to perfect his knowledge, not to learn his primary chapters in the text-book of clinical medicine. That is a position which is definitely recognised and allowed for in every well-conducted course of post-graduate study. At the Polyclinic, for example, it is a point which is always held well in view. There teaching is, as it should be in such an institution, illustrative, not dogmatic. But it is teaching that only goes to a certain limit. Actual bedside treatment cannot be, and is not, given. In the wards of the general hospitals the post-graduate practitioner has often to mingle with the students, and much of what he hears and sees is meant for the latter. With a few exceptions (among which we must rank the West London Hospital high) the subject of bedside treatment is the least illustrated item in the ordinary post-graduate course. The qualified practitioner desires to know how to treat his own patients, and the tendency is to show him how to treat patients in hospital—a very different thing.

The proper way out of this difficulty, which appears to us to be a real and not an imaginary one, is greater cohesion between the various post-graduate schools and associations, and relatively greater insistence on the importance of actual demonstrations at the bedside in selected cases. The ideal institution which one has in mind is an institution like the Polyclinic grafted on a small general hospital which is general in so far that it admits and treats all manner and variety of diseases, but special inasmuch as it only offers beds and treatment to selected cases. In other words the ideal method is to exercise in such a hospital the same careful discriminating selection that excludes from the clinical demonstration room at the Polyclinic merely " student " cases or matters which are not of actual practical interest. There appears to be no reason why such an institution should not be as successful as the Polyclinic is; there is no reason why the Polyclinic itself in course of time should not evolve such an institution. There are, we admit, difficulties in the way, for there are always difficulties in the way of realising any ideal; but they are not insurmountable. The financial one is, of course, the gravest, but ways and means may be found to overcome it. It is almost an anomaly that an institution like the Polyclinic, with a student-roll of which a university might reasonably be proud, should lack, for want of funds, the means of enormously increasing its usefulness and adding to the benefits its students may reap from it. An appeal to past and present students and to the profession generally, which is daily becoming more cognisant of the value and usefulness of graduate study, and finally to the philanthropic public, who would certainly give towards such an object were the whole case laid clearly before it, will surely enable the means to be found and the realisation to be achieved. We need not labour to point out the usefulness of such a scheme were it once realised. It would create, practically in the centre of London, a post-graduate institution which would be almost ideal, for it would enable men to treat patients and to watch and observe for themselves what they cannot at present do with the limited means at their command. It would enable the great mass of qualified medical workers who are at present scattered about in various parts of the Metropolis to conserve their energies. Finally, it would tend to bring together the various post-graduate courses, for it would interfere with no special class or series of demonstrations.

The work of perfecting and arranging such a scheme as we have here attempted to outline is one that properly and primarily should be initiated by the London Post-graduate Association. There are, we understand, changes pending in the organisation and work of this body, changes which have for their object the betterment of post-graduate study generally in the Metropolis. The Association speaks not on behalf of any special hospital or institution, and it is undesirable that it should limit or confine its energies to the advancement of one special section of the work of the post-graduate study movement. It will have to take into consideration the claims of every institution in London whereat special classes have been systematically arranged for the benefit of the qualified practitioner; but in the circumstances it appears that the Polyclinic, the most representative of our post-graduate institutions, the one with the largest following and the one which has undoubtedly done the pioneer work in this country, should have a prior claim when the subject of ideal extension is discussed.

When the Polyclinic was started one of its objects, as outlined in the prospectus, and as laid stress upon by Sir William Broadbent and other speakers at the festival dinner, was the advancement of medical science by the institution of special committees for research work on selected subjects. During the last few years this object has not been kept in view so strictly as it might have been. It is true the difficulties in the way of prosecuting original research in such an institution are greater in some ways than those which confront the inquirer at special and general hospitals. Nor, do we imagine, is the ardour for investigation so pronounced in graduate students as it is in men who are preparing for examinations or for the writing of epoch-making theses. Yet it is desirable that the institution as such should bear in mind what are the possibilities in this direction of encouraging and stimulatng orginal research. The material for investigation lies at hand, or is, at all events, readily accessible, and once the subjects have been definitely arranged, the workers will not be found wanting. Leprosy, yellow fever and tuberculosis, the subjects originally scheduled in the section that dealt with this branch of the Polyclinic's activities, are all of important interest, and all worthy the serious attention of the post-graduate student who has the time, the means and the inclination to devote himself to the pleasures of original research.

We venture to think that by systematically encouraging such research the Polyclinic and other post-graduate institutions in London may do much for the furtherance of medical science. But in order to provide definite encouragement it is necessary that the funds at the disposal of these institutions should be largely increased. There is wide scope, then, in this direction for the philanthropy of private individuals and public bodies who have shown themselves practically interested in the advancement of science by research in this country. The endowment of a few research scholarships at these post-graduate institutions will be productive of much good, and the arguments in favour of such encouragement are self-evident. Scholarships of this nature will give a special stimulus to research by the qualified practitioner—that is, by an observer who has perhaps a more matured judgment and a far greater conception of the practical value of one or other line of investigation than the unqualified student. Such research at such an institution should be primarily clinical, or, at least, its results should have a bearing upon the treatment and the elucidation of the cause of disease. Were such a scheme of special research instituted at the Polyclinic, we have no doubt that the collective reports of the various workers would in course of time add very greatly to our knowledge, and that such additions would be of immense practical benefit to the profession at large.

